# Bioinformatic validation and machine learning based exploration of B cells-related gene signatures in the context of strategies for precision therapy to acute myeloid leukemia

**DOI:** 10.1016/j.gendis.2025.101620

**Published:** 2025-04-11

**Authors:** Yongyu Chen, Xue Qiu, Jiehua Deng, Jianchao Ma, Jiansheng Huang, Yequan Lu, Ruilin He, Bin Liang

**Affiliations:** a Department of Hematology, Minzu Hospital of Guangxi Medical University, Nanning, Guangxi 530000, China; b Department of Cardiology, The First Affiliated Hospital of Guangxi Medical University, Nanning, Guangxi 530000, China; c Department of Respiratory and Critical Medicine, The Eighth Affiliated Hospital, Sun Yat-sen University, Shenzhen, Guangdong 518000, China; d Department of Orthopedics, Minzu Hospital of Guangxi Medical University, Nanning, Guangxi 530000, China; e Department of Respiratory, Nanxishan Hospital of Guangxi Zhuang Autonomous Region, Guilin, Guangxi 541000, China; f Department of Pain Medicine, The Second Affiliated Hospital of Guangxi Medical University, Nanning, Guangxi 530000, China; g Department of Gastroenterology, Minzu Hospital of Guangxi Medical University, Nanning, Guangxi 530000, China

Acute myeloid leukemia (AML) is a ubiquitous hematological cancer that originates from uncontrolled proliferation of bone marrow cells and presents distinct features in the population. Despite advances in chemotherapy and hematopoietic stem cell transplantation, AML remains a major challenge in improving patient survival. B cells play an important role in the immune monitoring, tumor microenvironment and therapeutic response of AML. In one aspect, AML cells can evade immune surveillance by a variety of mechanisms, including altering the expression of surface antigens, secreting immunosuppressive factors, or inducing the activity of immunosuppressive cells, which can recognize abnormal cell motility immune surveillance by impeding the production of antibodies. On the other hand, B cells and cytokines produced by them may play a supportive role in the pathogenesis and progression of AML. Accordingly, the functions of B cells and other immune cells of AML patients may be suppressed and thus may not be effective against AML cells. Therefore, this study aims to comprehensively explore the pathogenesis of B-cell-related genes in AML patients, explore the relationship between clinical outcomes of AML patients and tumor immune microenvironment characteristics, and provide ideas for precise treatment of AML patients.

In the study, we developed and validated a prognostic model associated with B cell-associated gene signature. Additionally, we preliminarily explored disease regulatory network construction, prediction of small molecule drugs, and drug sensitivity analysis of the prognostic genes. We found the prognostic risk profiles, established using five signature genes (SMPD3, MIR6774, PTPRS, SLC41A2, and UGCG) could effectively distinguish AML patients into subgroups with significant differences in both clinical characteristics and survival outcomes. The results indicated that there was a strong relationship between the prognostic models associated with B cell-related genes and the immune microenvironment. B cell-related genes could potentially serve as prognostic biomarkers for AML (Fig. 1).

**Figure 1:**
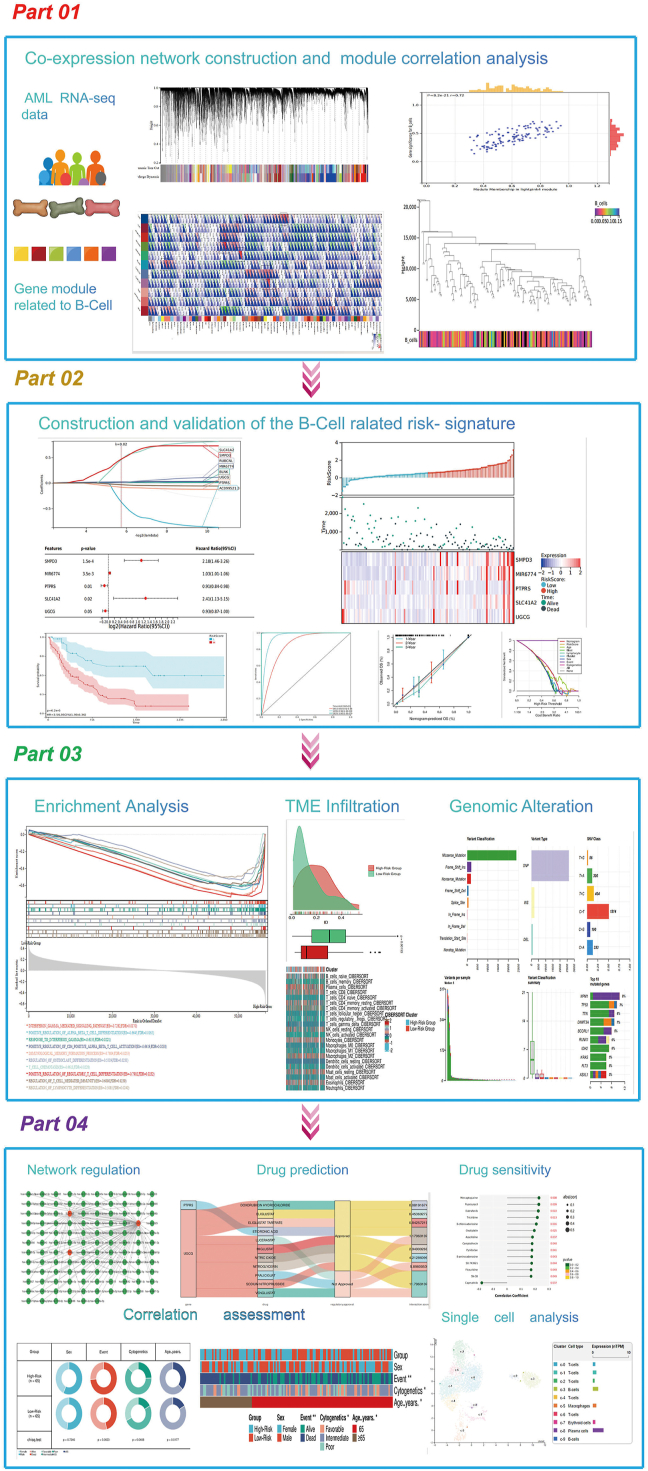
Flow chart of the study. This study consists of four parts: Part 1 “Construction”, Part 2 “Evaluation”, Part 3 “Characterization”, and Part 4 “Clinical Application”. The first part mainly describes the screening of genes significantly related to B cells using the WGCNA method. The second part mainly describes the use of Lasso and Cox methods to identify prognostic genes and establish a risk prediction model to verify its reliability. The third part mainly describes the potential pathogenesis of AML by comparing the differences in functional enrichment, gene mutations, immune microenvironment, and other aspects between high-risk and low-risk AML patients. The fourth part mainly describes the clinical application of establishing risk prediction models, such as searching for potential targeted therapeutic drugs and exploring the regulatory network of targeted genes.

We identified 5 prognostic genes using WGCNA, LASSO, and COX algorithms. The results showed that the prognostic risk profile established by these 5 characteristic genes (SMPD3, MIR6774, PTPRS, SLC41A2, and UGCG) could yield predicting results. To determine the validity of our predictive model, we performed a comparative analysis with other AML predictive models, and the results showed that the risk model constructed in this study was superior to other AML models in predicting 1-, 3-, and 5-year survival. These results indicated the potential of using WGCNA method to construct risk score of B-cell related genes, thus providing a theoretical reference for accurately evaluating the prognosis of patients (Fig.S1,S2,S3,S6,S7,S9).

Given that B cells play an important role in mediating the tumor microenvironment, we aimed to compare the tumor immune microenvironment of patients in the high/low risk groups and elucidate the underlying pathogenesis associated with immune function. Our results showed a significant prevalence of immune cells such as B cells, CD4+ T cells, macrophages, and myeloid dendritic cells in the low-risk group of AML. In contrast, NK cells and CD8+ T cells predominated in the AML high-risk group (Fig.S5,S8). Based on our analysis of immune cell infiltration and previous literature, we hypothesized that tumor cells may regulate the immune microenvironment in AML patients through a variety of mechanisms. There are two main ways of this regulation: first, tumor cells can induce immune suppression of immune cells according to their activity, resulting in changes in tumor microenvironment and promoting tumor progression; second, tumor cells can evade immune surveillance by reducing the number of B cells, thus realizing immune escape.

Gene mutation plays an important role in the development of acute myeloid leukemia. A better understanding of gene mutations in AML patients can help to more accurately predict disease progression and prognosis, and provide potential therapeutic targets for the development of new therapeutic strategies. In this study, we found that the frequencies of mutations in RUNX 1, ASXL 1, NPM 1, BCORL1, DNMT3A were relatively high (Fig. S4.). Based on the published results, we briefly summarize the effects of these genes on disease development. RUNX 1 is part of AML 1-ETO fusion protein, which can directly inhibit the transcription of tumor suppressor genes dependent on RUNX 1, destroy the differentiation of normal hematopoietic cells and promote the development of leukemia; on the other hand, RUNX 1 can inhibit the activity of some hematopoietic transcription factors and further destroy normal hematopoietic tissues. Previous studies have shown that wild-type ASXL 1 plays an important role in the maintenance of normal hematopoietic function. The deletion of ASXL 1 results in the arrest of differentiation of myeloid progenitors and the development of myeloid malignancies. Mutations in NPM 1 result in a stronger nuclear export signal than a nuclear localization signal, resulting in abnormal cytoplasmic localization of the mutated NPM 1 protein, which is thought to play a key role in leukogenesis. The b-cell lymphoma factor 6 co-suppressor (BCL6 co-suppressor, BCOR) is located on the X chromosome Xp 11.4 and contains 15 exons. BCORL1 (BCL6 co-suppressor such as 1) is a BCOR homolog, located on the X chromosome at Xq26.1, containing 12 exon. It has been found that BCOR /BCORL1 is involved in the proliferation and differentiation of myeloid cells, and plays an important role in transcriptional and epigenetic regulation. BCOR/BCORL1 mutations are common in myeloid tumors, such as acute myeloid tumors, and detectable mutation types include small insertions and deletions, frameshift mutations, nonsense mutations, and splice site mutations. Some studies have demonstrated poor prognosis in patients with myeloid tumors with BCOR/BCORL1 mutations. DNMT3A is a DNA methyltransferase that plays a key role in promoting hematopoietic cell differentiation. DNMT3A mutations significantly alter genomic methylation levels, resulting in altered patterns of gene expression and epigenetic regulation, leading to arrested differentiation and hyperproliferation of hematopoietic cells.

Finally, we attempted to explore the pathogenesis of the disease by studying five prognostic genes (SMPD3, MIR6774, PTPRS, SLC41A2, and UGCG) through previous literature reports. The SMPD3 gene plays a key role in cancer, particularly in the lipid remodeling of cancer cells. Overexpression of SMPD3 results in the accumulation of phosphatidylcholine (PC) of monounsaturated fatty acids (MUFAs), a change that correlates with tumor growth and invasiveness.^1^ The role of MIR6774 gene in cancer mainly reflects the signal pathway that may participate in the regulation of cell proliferation and apoptosis,^2^ thus promoting malignant transformation and invasiveness of cancer cells. The PTPRS gene affects the activity of the RAS pathway by regulating phosphorylation of extracellular signal-regulated kinases and nuclear translocation; in addition, the mutation status of PTPRS may affect the response to immune checkpoint inhibitors and prognosis.^3^ SLC41A2 gene plays a role through the combined action of MAPK and other key oncogenes in PI3K/AKT signaling pathway. UGCG is an enzyme located in the Golgi apparatus that is responsible for transferring udp-glucose molecules to ceramides to produce glucosylceramides, which are precursors of all complex glycolipids.^4^ Overexpression of UGCG affects cell signaling by altering the lipid composition of the cell membrane,^5^ thereby promoting tumor growth and invasiveness. Taken together, these results provide a basis for future precise immunotherapy of AML patients.

## CRediT authorship contribution statement

**Yongyu Chen:** Writing – original draft, Resources, Project administration, Methodology, Investigation, Data curation. **Xue Qiu:** Writing – original draft, Visualization, Validation, Software, Methodology, Investigation. **Jiehua Deng:** Writing – original draft, Visualization. **Jianchao Ma:** Resources, Validation, Visualization. **Jiansheng Huang:** Resources, Investigation, Formal analysis, Data curation. **Yequan Lu:** Investigation, Methodology, Resources, Software. **Ruilin He:** Project administration, Supervision, Visualization, Writing – review & editing. **Bin Liang:** Software, Resources, Project administration, Writing – review & editing.

## Conflict of interests

The authors declared no conflict of interests.
