# Supplementary material for: Bioinformatic validation and machine learning based exploration of B cells-related gene signatures in the context of strategies for precision therapy to acute myeloid leukemia
Source: Genes Dis. 2025 Apr 11;13(1):101620. doi: 10.1016/j.gendis.2025.101620 (PMC12624567; doi:10.1016/j.gendis.2025.101620)
Supplement: Multimedia component 1 [file mmc1.docx]

**Materials and method**

**Data source and access**

For the training set, RNA sequencing transcriptome data and corresponding baseline information were retrieved for AML patients from the TCGA repository (https://portal.gdc.cancer.gov/). Patient records with comprehensive transcriptional and clinical profiles from dataset GSE37642 in the Gene Expression Omnibus (GEO) database (https://www.ncbi.nlm.nih.gov/geo/) were downloaded to form the validation set. Samples from TCGA were selected based on these criteria: 1) confirmed AML diagnosis; 2) accessibility of transcriptome data; and 3) availability of overall survival details and associated clinical parameters. This selection process yielded 130 bone marrow samples from the TCGA for analysis. The microarray data preprocessing steps for the GSE37642 cohort included: 1) omitting samples without adequate follow-up; 2) translating probe IDs to the Gene Symbol format; 3) removing probes that mapped to multiple genes; and 4) averaging values when multiple probes were linked to a single gene. Following these procedures, 140 bone marrow samples from the GSE37642 cohort were incorporated into the study.

**Co-expression network construction and module correlation analysis**

The Weighted Gene Co-expression Network Analysis (WGCNA) was employed to establish a co-expression network. This involved creating a weighted adjacency matrix using the formula amn = |cmn|^β， where cmn represents the Pearson correlation coefficient between genes m and n, and amn signifies the adjacency between these genes. The parameter β serves as a soft threshold that underscores strong gene interrelations, ensuring a goodness of fit exceeding 0.8. This is achieved by selecting an appropriate soft threshold parameter to form a scale-free co-expression network. Furthermore, the WGCNA technique was applied to transform immune cell trait data into color codes, resulting in a heatmap that illustrates the correlation between gene modules and immune characteristics.

**Construction and evaluation of risk signature based on hub module**

The Least Absolute Shrinkage and Selection Operator (LASSO) in conjunction with Cox regression analyses represent prevalent statistical methods. These approaches integrate variable selection with regularization, enhancing both the predictive accuracy and the comprehensibility of the derived statistical models. In this research, both LASSO and Cox regression were employed to pinpoint independent risk factors associated with Acute Myeloid Leukemia (AML). The R programming package "RMS" was utilized to develop prognostic nomograms, evaluating their utility in forecasting outcomes. The potential for devising a risk model across varying clinical attributes was then examined through Kaplan-Meier survival analysis. The precision of the proposed model was gauged by generating a receiver operating characteristic curve (ROC). The external validity of the risk index was confirmed using dataset GSE37642. Integrating clinical traits with the risk index, a nomogram was formulated to anticipate AML prognosis.

**Functional Enrichment Analysis**

Through the utilization of the Gene Ontology (GO) database, a functional enrichment analysis was conducted. This analysis aimed to uncover the signaling pathways and biological impacts that are prevalent among the set of genes associated with B cells. A P-value threshold of 0.05 was established for this purpose. To further validate these findings, a Gene Set Enrichment Analysis (GSEA) was undertaken using the MSigDB collection (c2.cp.kegg.v7.4.symbols.gmt). This step assessed whether notable variations existed in the gene sets expressed between the high / low-risk groups. For the results to be considered statistically significant, P-values had to be less than 0.05 and the False Discovery Rate (FDR) had to be below 0.25.

**Somatic mutation analysis and characterization of the immune landscapes** **between subgroups**

The somatic mutation profiles of AML samples were retrieved from the TCGA database, and distinct variations in these profiles between two sample subgroups were illustrated using a waterfall plot. To investigate immune cell penetration and to discern disparities in the tumor microenvironment across these subgroups, an immune infiltration analysis was conducted. This analysis employed several algorithms, including XCell, quanTlseq, MCPCOUNTER, and CIBERSORT.

**Construction of miRNA gene and TF gene regulatory network, identification of potential targeted drugs, and drug sensitivity analysis**

The Networkanalyst platform (accessible at https://www.networkanalyst.ca) was utilized to identify microRNAs and transcription factors that interact with the five identified prognostic genes. This tool also facilitated the construction of regulatory networks, elucidating potential interactions between microRNAs and genes, as well as between transcription factors and genes. For exploring potential targeted therapeutics, the Drug-Gene Interaction Database (available at https://dgidb.org) was consulted to uncover associations between drugs and genes. Recognizing that many patients with acute myeloid leukemia often exhibit poor responses to drug treatments, a drug sensitivity analysis was undertaken in this study. The goal was to offer more promising therapeutic options for those battling acute myeloid leukemia. This drug sensitivity analysis drew from the extensive drug screening dataset available at https://zenodo.org/records/7274740.

**Molecular docking**

Molecular docking is a computational chemical method for studying interactions between molecules (e. g. drug) and molecules (e. g. target protein). To detail the binding mode of small molecules on the surface of biological macromolecules to predict possible binding modes and binding abilities between them. We were selected separately from the RCSB PDB database (https://www.rcsb.org/) and the PubChem database (https://pubchem.ncbi.nlm.nih.gov). Structural information of target proteins and drugs was downloaded by the above database and was visualized available at an online drug discovery platform (mcul.com) for the 3D / 2D structure.

**Single-cell expression analysis and Subcellular localization of biomarkers**

Utilizing the Human Protein Atlas database (accessible at https://www.proteinatlas.org/), single-cell and transcriptional data were employed to examine the expression levels of biomarkers in bone marrow cells. Additionally, by referencing the COMPARTMENTS database (found at https://compartments.jensenlab.org/), we further anticipated the subcellular positioning of these biomarker proteins.

**Statistical analysis**

The assessment of continuous variables across two distinct groups was conducted utilizing the two-sample t-test, while the chi-square test was employed for categorical data analysis. Patient survival rates were estimated and survival curves plotted using the Kaplan-Meier methodology in conjunction with the log-rank test. Statistical significance was determined by a P value below 0.05.

**Result**

**Identification of hub module associated with B cells infiltration by WGCNA**

The dynamic shear tree method was employed to conduct clustering analysis, with a shear height of 20,000 serving as the threshold for shearing. B cell data were then transposed into color codes and depicted in both a dendrogram and a distinctive heat map (**Fig.S1A).** The selected genes were utilized to form a scale-free network by determining the connection strengths. The determination of the scale-free topology model was guided by a scale-free R^^2^ ranging from 0 to 1. **Fig.S1B** illustrates the correlation between the goodness of fit (R^^2^) and various soft thresholds; the red line denotes an R^^2^ value of 0.85, where an R^^2^ near or exceeding 0.9 is deemed satisfactory. **Fig.S1C** presents the association between the mean number of connections and different soft thresholds. Notably, when the soft threshold β equals 8, the R^^2^ value coincides with a network average connectivity approaching zero, and the gene distribution aligns with that of a scale-free network.

Genes exhibiting co-expression traits were categorized by color, employing a shear height of 0.25 to create a module clustering diagram (**Fig.S1D**). In this representation, each leaf signifies an individual gene, while each branch embodies a co-expression module. The correlation between these modules and immune cell attributes was then determined, leading to the creation of a module-feature association plot. This analysis revealed that the B cells exhibited a significant positive correlation with the lightpink4 module, which encompassed 16 genes. Consequently, the lightpink4 module was designated as the central module, and genes from this module were isolated for further investigation (**Fig.S1E**).





**Fig.S1 WGCNA analysis**. (A) Tree diagram and feature heat map. (B) Relationship between the goodness of fit and different soft thresholds. (C) Relationship between the average number of connections and different soft thresholds. (D) Module clustering diagram. (E) Association diagram between gene modules and immune cell characteristics.

**Construction of prognostic signature based on genes of lightpink4 module**

LASSO regression was performed to pinpoint genes within the lightpink4 module associated with the overall survival of AML patients. Eight genes were identified and subsequently analyzed using univariate Cox regression analysis (**Fig.S2A**). To refine stable variables and prevent model overfitting, five genes identified through univariate Cox regression were assessed (**Fig.S2B**). This process resulted in five prognostic genes that were utilized to develop a risk signature. Based on the median risk score threshold, AML patients were categorized into high-risk and low-risk groups. Notably, the prognosis for the high-risk group was less favorable compared to the low-risk group (**Fig.S2C**). Integrating the risk signature with clinical attributes from the TCGA cohort, a nomogram was constructed to predict patient survival (**Fig.S2D**). The calibration curves indicated that the predicted survival rates at 1, 3, and 5 years aligned closely with the actual survival rates (**Fig.S2E**). Kaplan-Meier survival analysis revealed a significant correlation between the OS of AML patients and their risk scores, with the high-risk group exhibiting notably reduced OS, suggesting that an elevated risk score is a marker of poor prognosis for AML patients. The ROC curve areas for 1, 3, and 5 years were 0.85, 0.96, and 1.00, respectively, underscoring the reliability of the risk signature in predicting OS among AML patients (**Fig.S2F**). Validation using the GEO dataset yielded congruent results (**Fig.S2G**). To ascertain the efficacy of our predictive model, a comparative analysis with other AML predictive models revealed that WGCNA method-based model, which is closely linked to B cells, outperformed other AML models in predicting 1-year, 3-year, and 5-year survival rates (**Fig.S2H**).





**Fig.S2** Construction and validation of the B Cells-risk signature. (A) Lasso Cox analysis; (B) Univariate Cox analysis to evaluate the specific prognostic value of B Cells-related genes; (C) Risk scores distribution, survival status of each patient, and heatmaps of prognostic 5-gene signature in TCGA database; (D-E) Construction of the monogram、Calibration and Decision Curve Analysis diagram. Time-dependent ROC curves and Kaplan-Meier analysis in the TCGA cohort (F) and GSE37642 cohort (G). (H) Performance comparison between B Cells-risk signature and other signatures.

**Enrichment analysis and identification of signaling pathways**

To elucidate the molecular mechanisms underlying prognosis, we initially identified signaling pathways associated with B cell genes. Our analysis revealed that in the biological processes category, B cell-related genes were enriched in various macromolecular synthesis processes, including ceramide biosynthesis, glycosphingolipid metabolism, and ceramide metabolism. Additionally, in the cellular component categories, B cell-related genes were found to be enriched in organelles such as the Golgi apparatus and synaptic vesicles. In terms of molecular function categories, B cell-related genes played roles closely related to life metabolism, such as transferases and hydrolases (**Fig.S3A-C**).

GSEA indicated that immune-related pathways, including immune cell differentiation and activation, were significantly enriched in the high-risk group. Conversely, in patients with low-risk subgroup, pathways related to biomolecular synthesis and metabolism were significantly enriched (**Fig.S3D-E**). GSEA analysis of these five prognostic genes showed that in the high-risk AML population, these genes were involved in multiple biological processes related to immune and metabolic functions (**Fig.S9A**).





**Fig.S3 Exploration of the pathogenesis of AML. (**A, B, C) Biological Process, Cellular Component and Molecular Function analysis of 5-gene signature. (D-E) GSEA analysis identifies potential signaling pathways between subtypes.

**Somatic mutational analysis**

Initially, we determined the chromosomal locations of the five prognostic genes (**Fig.S4A**). Subsequently, we conducted an analysis to compare the pathways predominantly enriched in AML categorized as high or low-risk group. Our findings revealed distinct somatic mutation profiles among AML subgroups, noting that genetic mutations in NPM1, TP53, DNMT3A, BCORL1, RUNX1, and IDH2 were more prevalent in somatic mutations among AML patients. However, the relative frequencies of these mutations differed across subgroups. Signaling pathways such as RTK-RAS, WNT, TP53, and Hippo were significantly enriched in various risk groups, suggesting potential key pathogenic pathways and offering a theoretical foundation for precision medicine approaches, including subsequent targeted therapy (**Fig.S4B-G**). Additionally, we discovered that the genetic mutations in these AML patients with somatic mutations were primarily of the nonsense mutation type and manifested as single nucleotide polymorphism variations (**Fig.S4H**).





**Fig.S4** **Analysis of gene mutations.** (A) The positions of 5 prognostic genes on chromosomes; (B-C) Common pathogenic pathways and mutated genes in the high-risk group; (D-E) Common pathogenic pathways and mutated genes in low-risk group. The common types and forms of genetic mutations in AML patients with somatic mutations (F-H).

**Landscapes of the tumor microenvironment**

Considering the significant role of B cells in the anti-tumor immune response, we conducted a thorough investigation of the tumor microenvironment across different AML patient subgroups using various algorithms (CIBERSORT, MCPCOUNTER, quanTlseq, and XCell). Initially, we compared the expression levels of diverse immune cell types among the subgroups. In the low-risk AML subgroup, we observed a higher proportion of immune cells such as B cells, CD4+ T cells, macrophages, and myeloid dendritic cells; whereas in the high-risk subgroup, NK cells, Treg cells, and CD8+ T cells exhibited elevated expression levels (**Fig.S5 A-D**). Furthermore, upon comparing the scores between the high and low-risk groups, we found that low-risk AML patients exhibited higher immune scores and microenvironment scores, while high-risk AML patients displayed higher matrix scores, with these scores showing statistically significant differences. These findings underscore the close association between B cell risk characteristics and the tumor microenvironment (**Fig.S5 E-G**).





**Fig.S5** Immune landscapes between subgroups. (A-D) Relative proportion of immune cell infiltration in subgroups by XCell、quanTlseq、MCPCOUNTER and CIBERSORT algorithm.(E-G) Comparison of ImmuneScore、Microenvironment Score and Stroma Score in subgroups.

**Clinical application of the risk model**

To evaluate the effectiveness of developing risk signatures related to B cells, we initially examined the correlation between the risk score and the expression levels of various immune cells. Notably, we discovered a significant positive correlation between the risk score and the expression levels of certain immune cells, such as monocytic cells, myeloid dendritic cells, and cytotoxic lymphocytes. In contrast, the expression levels of B cells exhibited an inverse relationship with the risk score. Overall, the five prognostic genes were found to be closely linked to most types of immune cells, particularly monocytic cells, myeloid dendritic cells, and B cells (**Fig.S6 A1-A2**). Subsequently, we created a comprehensive heatmap and a donut chart to illustrate the differences in clinical information between AML patients in the high and low-risk groups, thereby demonstrating the successful establishment of risk signatures for B cell-related genes and their potential clinical relevance (**Fig.S6 B/D**). Finally, we conducted a subgroup survival analysis of AML patients based on criteria such as sex, age, prognosis, and event stratification to validate the reliability of this predictive model (**Fig.S6 F1-F5**). Additionally, we compared the expression patterns of these five prognostic genes between high and low-risk AML subgroups (**Fig.S9 B**).



**Fig.S6** Clinical application of the risk scores. (A1) Correlation analysis of risk scores and expression of various types of immune cells; (A2) Correlation analysis of 5-gene signature and expression of various types of immune cells; (B) Complex heatmaps display differences in the distribution of clinical data among high/low-risk groups patients; (C) miRNA-gene and transcription factor-gene regulatory network; (D) Different comparisons of number of patients in age, sex, cytogenetics and survival events between subgroups; (E) Identification of the targeted drugs for prognosis genes; (F1-5) Subgroup survival analysis.

**Regulatory network construction, small-molecule drug prediction, drug sensitivity analysis, and molecular docking of the prognostic genes**

The miRNA is a type of endogenous short non-coding RNA that can effectively mediate mRNA degradation, and the transcription factors (TFs) can bind to specific DNA sequences and play a role in regulating gene expression. To gain a deeper understanding of the pathogenesis of AML, we predicted miRNA and transcription factors that regulate prognostic genes and constructed miRNA-gene and transcription factor-gene regulatory networks using NetworkAnalyst software. A total of nearly 40 TF and 113 miRNA showed complex regulatory relationships with prognostic genes (**Fig.S6 C1-C2**). Next, we focused on exploring potential therapeutic agents for AML. First, we used the DGIdb database to explore the interaction network of potential targeted drugs-prognostic genes, providing a theoretical reference (**Fig.S6 E**) for the subsequent precise treatment of AML. At the same time, we performed a drug sensitivity analysis of these prognostic genes. The results showed that chemotherapeutic drugs such as Daporinad, Camptothecin, and Nilotinib have good therapeutic effects on the SL41A2 gene. For the SMPD3 gene, chemotherapeutic agents such as Lomustine and Mechlorethamine are more sensitive to it and show therapeutic potential. For the UGCG gene, potential therapeutic agents such as Mercaptopurine, Fluorouracil, and Dabrafenib have shown sensitive therapeutic effects. However, for the PTPRS gene, chemotherapeutic drugs such as StemRegenin 1 and Tazemetostat are more sensitive to them. Overall, the above findings facilitate potential treatment strategies for AML patients (**Fig.S7 A-D**). We also additionally show a schematic diagram of the 3D/2D molecular docking of SMPD3 and PTPRS genes with two of the targeted therapeutic drugs, which is conducive to laying the theoretical basis for subsequent drug development technologies (**Fig.S7 E-H**).





**Fig.S7 Exploring potential drugs**. **(**A-D) Drug sensitivity analysis of the prognostic genes; (E-H) Molecular docking techniques clearly demonstrate the chemical structure binding details of molecular PTPRS with potential targeted therapeutic drugs StemRegenin 1 and Tazemetostat, as well as molecular SMPD3 with potential targeted therapeutic drugs Lomustine and Pevonedistat.

**Single-cell analysis and subcellular localization of the prognostic genes**

To more precisely delineate the expression patterns of prognostic genes in human bone marrow tissue, we employed scRNA sequencing utilizing the HPA database to discern the cell populations present within the bone marrow. Through clustering, nine distinct myeloid cell subsets were identified, as depicted in the UMAP plot. Further analysis uncovered that PTPRS is predominantly expressed in T and B cells, SL41A2 in plasma cells and B cells, and SMPD3 in both T and B cells. Notably, UGCG exhibited high expression levels in macrophages, T cells, B cells, and plasma cells (**Fig.S8A**). Proteins serve as indicators of diverse biological functions, which are contingent upon their cellular localization. By referencing the Compartments database, we further anticipated the subcellular localization of these prognostic proteins. PTPRS is primarily localized to the distal axon, SL41A2 to the plasma membrane, SMPD3 to the extracellular exosome, and UGCG to the lysosome (**Fig.S8B**).





**Fig.S8** **Mapping of the targeted genes.** (A) Single-cell expression analysis (B) and subcellular localization analysis of hub genes





**Fig.S9** **Functional analysis and expression of the targeted genes**. (A) Gene set enrichment analysis for the five-gene signature; （B）Expressions of the five genes of the prognostic signature in acute myeloid leukemia.
