# Supplementary material for: Bioinformatic validation and machine learning based exploration of B cells-related gene signatures in the context of strategies for precision therapy to acute myeloid leukemia
Source: Genes Dis. 2025 Apr 11;13(1):101620. doi: 10.1016/j.gendis.2025.101620 (PMC12624567; doi:10.1016/j.gendis.2025.101620)
Supplement: Multimedia component 2 [file mmc2.docx]

**legend**

**Figure 1** Flow chart of the study. This study consists of four parts: Part 1 "Construction", Part 2 "Evaluation", Part 3 "Characterization", and Part 4 "Clinical Application". The first part mainly describes the screening of genes significantly related to B cells using the WGCNA method. The second part mainly describes the use of Lasso and Cox methods to identify prognostic genes and establish a risk prediction model to verify its reliability. The third part mainly describes the potential pathogenesis of AML by comparing the differences in functional enrichment, gene mutations, immune microenvironment, and other aspects between high-risk and low-risk AML patients. The fourth part mainly describes the clinical application of establishing risk prediction models, such as searching for potential targeted therapeutic drugs and exploring the regulatory network of targeted genes.

**Fig.S1** WGCNA analysis. (A) Tree diagram and feature heat map. (B) Relationship between the goodness of fit and different soft thresholds. (C) Relationship between the average number of connections and different soft thresholds. (D) Module clustering diagram. (E) Association diagram between gene modules and immune cell characteristics.

**Fig.S2** Construction and validation of the B Cells-risk signature. (A) Lasso Cox analysis; (B) Univariate Cox analysis to evaluate the specific prognostic value of B Cells-related genes; (C) Risk scores distribution, survival status of each patient, and heatmaps of prognostic 5-gene signature in TCGA database; (D-E) Construction of the monogram、Calibration and Decision Curve Analysis diagram. Time-dependent ROC curves and Kaplan-Meier analysis in the TCGA cohort (F) and GSE37642 cohort (G). (H) Performance comparison between B Cells-risk signature and other signatures.

**Fig.S3** Exploration of the pathogenesis of AML**. (**A, B, C) Biological Process, Cellular Component and Molecular Function analysis of 5-gene signature. (D-E) GSEA analysis identifies potential signaling pathways between subtypes.

**Fig.S4** Analysis of gene mutations. (A) The positions of 5 prognostic genes on chromosomes; (B-C) Common pathogenic pathways and mutated genes in the high-risk group AML; (D-E) Common pathogenic pathways and mutated genes in low-risk. The common types and forms of genetic mutations in AML patients with somatic mutations (F-H).

**Fig.S5** Immune landscapes between subgroups. (A-D) Relative proportion of immune cell infiltration in subgroups by XCell、quanTlseq、MCPCOUNTER and CIBERSORT algorithm.(E-G) Comparison of ImmuneScore、Microenvironment Score and Stroma Score in subgroups.

**Fig.S6** Clinical application of the risk scores. (A1) Correlation analysis of risk scores and expression of various types of immune cells; (A2) Correlation analysis of 5-gene signature and expression of various types of immune cells; (B) Complex heatmaps display differences in the distribution of clinical data among high/low-risk groups patients; (C) miRNA-gene and transcription factor-gene regulatory network; (D) Different comparisons of number of patients in age, sex, cytogenetics and survival events between subgroups; (E) Identification of the targeted drugs for prognosis genes; (F1-5) Subgroup survival analysis.

**Fig.S7** Exploring potential drugs. **(**A-D) Drug sensitivity analysis of the prognostic genes; (E-H) Molecular docking techniques clearly demonstrate the chemical structure binding details of molecular PTPRS with potential targeted therapeutic drugs StemRegenin 1 and Tazemetostat, as well as molecular SMPD3 with potential targeted therapeutic drugs Lomustine and Pevonedistat.

**Fig.S8** Mapping of the targeted genes**.** (A) Single-cell expression analysis (B) and subcellular localization analysis of hub genes

**Fig.S9** Functional analysis and expression of the targeted genes. (A) Gene set enrichment analysis for the five-gene signature; （B）Expressions of the five genes of the prognostic signature in acute myeloid leukemia.
